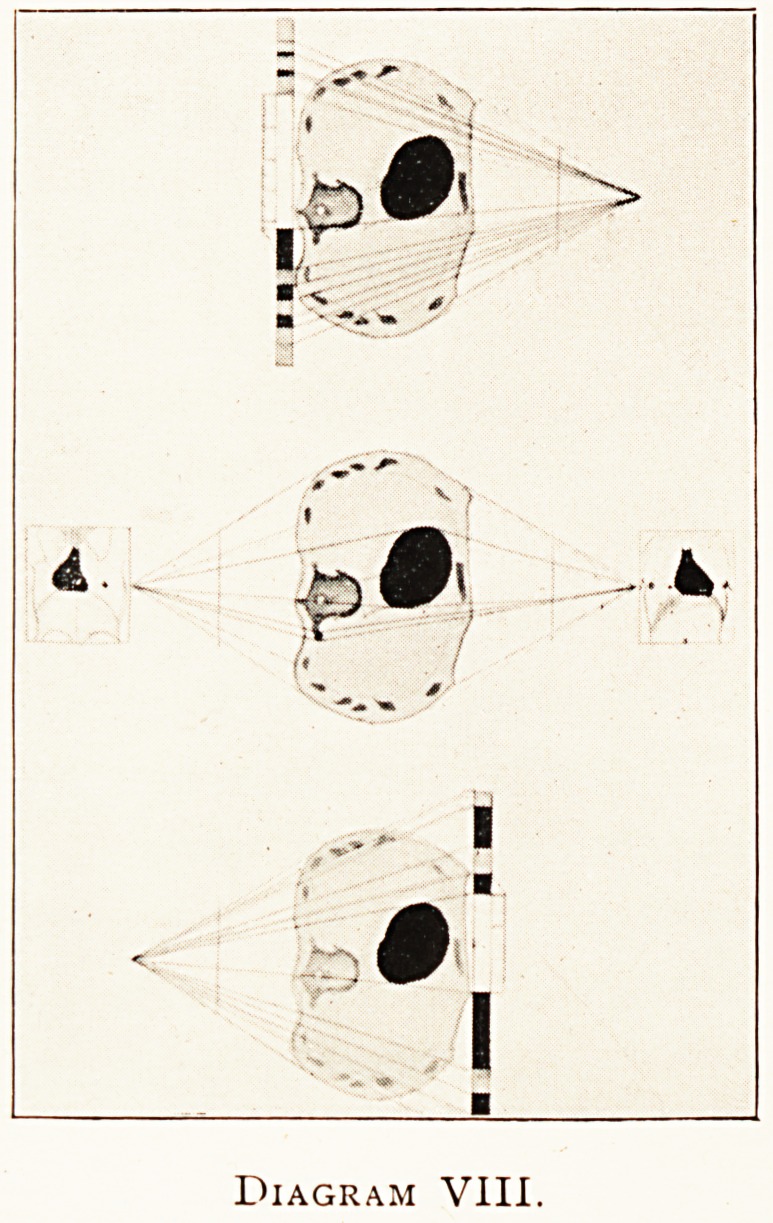# The Right and the Wrong Side of the X-Ray Picture; Has a Mistake Been Made?
1Read to the Röntgen Society of London, January 2nd, 1912.


**Published:** 1912-09

**Authors:** W. Cotton


### THE RIGHT AND THE WRONG SIDE OF THE X-RAY PICTURE; HAS A MISTAKE BEEN MADE?[1]

BY

W. COTTON, M.A., M.D.

---

A FLUORESCENT screen can be viewed practically only from the side remote from the focus tube in action, while the plate and even the print as a rule are transparent enough to be viewed at any other time from either side. The right side of any picture made by rays diverging from a point is the side towards which lies the correct station point of the observer's eye, otherwise known as the centre of projection. It is the only point from which, looking towards the object, it is possible to see all the parts of the picture of an object in the same directions and under the same angles as the corresponding parts in the object itself would subtend to the eye if the object were visible, point for point. The wrong side of any such picture made by radial projection is the side remote from the centre of projection, from no point towards which side can an eye ever see the object and its picture thus wholly in line, or as it is called "in perspective." It is the aim of the present paper to prove by example and experiment—so far as experiments on the drawing-board can be regarded as such—that the original station point of the X-ray centre of emission of the focus tube (in regard to the object depicted, and the X-ray picture thereof at the time of exposure) is the only correct station point of the interpreter's eye, when he seeks

[1] Read to the Röntgen Society of London, January 2nd, 1912.

to gather the greatest possible amount of reliable information from any given single X-ray picture. I hope also by the way to demonstrate some of the more immediate practical consequences of a highly paradoxical thesis, to which, since I first suggested it early in 1901, I have been unable, so far as I know, to win one single medical convert.[1]

In photography the centre of projection lies between the object to be taken and the picture plane ; in radiography and ordinary shadow projection from a near point the object lies between the picture plane and the centre of projection, while in ordinary drawing the picture plane is actually or mentally kept between the centre of projection and the object to be drawn. Practically as the case may be, the centre of projection is the optical centre of the camera lenses or the pinhole of a pinhole camera, or it is the X-ray centre of emission of the focus tube, or a small luminous flame, and in the case of an ordinary drawing it is the optical centre of the draughtsman's refractive media of one or other eye, the eye itself being a small camera.

When the picture plane (as in ordinary drawing) is between the object and the centre of projection, it may be conveniently spoken of as a graphic plane, the image being smaller than the object as a rule. In the case of radiography or ordinary shadow projection we may speak of the picture plane on the far side of the object from the centre of projection as a diagraphic plane, the shadow image thereon being larger than any corresponding parallel linear dimension in the object, except where the object comes in contact with the picture plane: there the image and object are of equal extent. A photographic plane may be erected anywhere, even virtually through the object to be taken, and may be of any size in relation to the object to be taken from the microscopic photograph of the planet Mars in the head of a breastpin, to the enlarged lantern slide projected on a white sheet of the photomicrograph of a cheese mite. Thus by photographic means we may enlarge the image on a graphic to one on a diagraphic plane, or we may similarly reduce the image on a diagraphic plane to any suitable graphic plane, say to lantern slide size. But the terms graphic and diagraphic are useful ones as long as we remember that a diagraphic plane is a plane of photographic enlargement; while a graphic plane is one of photographic diminution; some day no radiographic laboratory will be considered complete without a reduction camera. It is perhaps as well to mention here that in the case of any given object the various planes are supposed to be parallel to each other; and in the course of picture making the centre of projection, whatever it may be, is kept steady in the same position in regard to the object.

In Diagram I, PD is part of the face of an X-ray photographic plate with film towards O according to the prevailing practice, or the back of an X-ray fluorescent screen or of an X-ray print

DIAGRAM I.

supposed semi-transparent; O is the original station point of the X-ray centre of emission in regard to object and to PD. M and N are any two points of an object nearer PD than O and not in contact with PD; and m and n form the X-ray projection or shadow-image of M and N on PD. O′ is the station point of an observer's eye on what I call the wrong side of the X-ray picture plane, namely on the side remote from the X-ray centre of emission O. At O′ the shadow on the face of the fluorescent screen (or visible from the glass side of the X-ray photographic plate or on the face or film side of the X-ray print) m n is visible in different directions from what the corresponding points in the object M and N would appear in, namely in directions O′m and O′M, and O′N and O′n. To facilitate vision of the objects M and N there is represented an oval window in the semi-transparent plane PD. The observer may look from where he pleases on either side of PD, but until he places his eye at O looking towards PD he can never see either simultaneously or in succession m in the same direction or under the same angle mON as M appears in, nor n in the same direction or under the same angle nOM as N appears in. Now as the two straight lines OMm and ONn intersect only at one point O, O is the only point at which the observer's eye will see the object and its image in perspective. But O is the original station point of the X-ray centre of emission, therefore the original station point of the X-ray centre of emission of the focus tube (in regard to the object depicted, and the X-ray picture thereof at the time of exposure) is the only correct station point of the interpreter's eye. Q.E.D.

Owing to the essential ambiguity as to near and far of all shadow projection—so that the picturesque effect of the same in the case of objects whose internal structure is unknown is highly fallacious—a correct determination of the true *direction* in the object of the parts depicted is, perhaps, the main function of an X-ray picture (Diagram II). [2]

In the top figure the focus tube is shown in action with the centre of its target at the O of the last diagram ; on the far side of the fluorescent screen is shown an observer with his eye at a likely point, namely a point diametrically opposite the centre of projection, and at an equal distance from the picture, at what is called the corresponding station point to the picture plane. In this figure, and in the next below, it is evident that none of the seven points of the object will be seen on the face of the screen in the same direction as they would appear under in the object if visible from the corresponding station point, except one point that happens to lie in contact with the screen, and another which happens to lie in the axis joining the original station point with the corresponding station point. In the second figure the observer on the left of the diagram is contemplating

Diagram II.

Diagram III.

Diagram IV.

Diagram V.

from the corresponding station point the face of an X-ray print (or it might be the glass side of the X-ray negative), while on the right side of the diagram the observer is contemplating what may be either the film side of a developed glass negative according to prevailing usage, or the back of an X-ray print therefrom, supposed to be semi-transparent. In the third figure both observers are viewing the original object itself from opposite sides, in the same position thereto as in the first or second figures. In the fourth or lowest figure the observers are viewing the actual object from opposite sides of it; but while the observer on the right retains his original station point, the observer on the left has taken another likely station point on the axis, namely a point corresponding to the opposite mean distance from the object. In this figure I have indicated graphic planes in relation to both observers. No doubt there are other special cases not represented in the diagram, but it will be found that under no circumstances will an observer on the side of the X-ray picture plane remote from the original station point ever see the shadow picture wholly in perspective with the object throwing the shadow, while the observer with his eye in the original station point of the X-ray centre of emission in regard to object and X-ray picture plane will always continue to do so. But from what I call the wrong side of the picture plane, the observer can see only the shadows on the window blind; if provided with a pistol he must aim wide of his object (with the two exceptions I have indicated) and miss his mark: what he ought to do is to come out of the street and take sights through the key-hole.

In comparing shadow pictures in the matter of outlines with photographs and drawings of the same object from the same centre of projection, it is convenient to take some very definite shaped body at once X-rayable and visible, *e.g.* a right-handed cube with its back turned towards the centre of projection, made in a sort of open framework of opaque metal and painted black (Diagram III). In the top figure of this diagram are represented some of the photographic relations; on the extreme right is shown the inverted image as it would appear on the focusing screen of a camera; the next image on the left is an erect photographic print of the same size; the third image on the left is an enlarged photographic print. To avoid considering the perplexing subject of focusing by a lens at different distances, I have represented the camera as a very efficient pinhole one, the pinhole being further, as a matter of fact, closely comparable in size to the bombarded area on the target of the focus tube. In this figure is also indicated in outline an enlarging photographic camera. The second figure represents ordinary shadow projection by means of a small luminous flame. The third figure shows the image thrown by X-rays upon a fluoroscope supposed to be semi-transparent, or it might be an X-ray print illuminated from the left and showing through its paper back. The fourth figure shows an X-ray developed glass plate with its film side turned towards the focus tube as it actually lay at the period of exposure. The lowest figure shows the draughtsman finishing his task.

While Diagram III represents picture making, its continuation, Diagram IV, represents mainly picture viewing. The top figure represents simple vision of the object directly; the next figure the contemplation of an X-ray print through its back; the third the inspection of an X-ray negative from its face; the fourth shows the viewing of a finished drawing, or it might be a photographic print; while the lowest figure is a diagrammatic combination of the other cases, with an attempt at a rude dissection to expose the ocular camera itself.

Under the conditions set forth, all the images shown in the last two diagrams are similar in *form*, and are all in perspective register with each other and with the object, but they differ here and there in size and *tone*. Some are black on white, others are white on black. Photographic prints and the photographic picture on the focusing screen, the picture on the fluorescent screen, the ordinary shadow and the drawing as a rule are all black on white, what are called "positives," from whichever side of the picture plane they are viewed: the shadows (or the interruption by opaque bodies of the luminous agent) are black, while the high lights are white, so that where the luminous agent freely passes (by transmission in the case of X-rays, by reflection as a rule in the case of ordinary light) black on white is the correct arrangeemnt of tone. The photographic plate when developed, and the X-ray photographic plate when developed and suitably illuminated, are white on black—an incorrect arrangement of tone—what are called "negatives," from whichever side they are viewed. Correct *form*, therefore, depends not on *tone*, but on viewing the photographic or other positive or negative from the correct side of the picture. The analogy between the photographic and the X-ray print is correct as to *tone* only, but entirely misleading in the much more important matter of *form*. The X-ray negative viewed from its film side is the only true analogue of the photographic print as to *form*.

The next diagram (V) illustrates probably one of the most important points in a thorny subject, and certainly one of the hardest to explain and understand. In the two previous diagrams the object was a right-handed cube with its back towards the centre of projection and station point of the eye. When we view a right-handed cube of this kind in a plane mirror, we see a left handed cube, and vice versa. In the top figure of the present diagram is drawn an undifferentiated cube with its radiographic negative-image and print; its top and bottom, and back and front, and two sides are all indifferently alike. In the second figure is shown a right handed cube with its back towards the radiographic centre as in previous diagram; in the third figure there is as object a right-handed cube with its face turned towards the focus tube; in the next figure is drawn a left-handed cube with its face next the radiographic plane; and in the lowest figure the object is a left-handed cube with its face towards the radiographic centre. In the case of these last four objects are shown the X-ray negative (white on black) images, and the print corresponding to each (black on white). Let us call these negative images of the figured cubes from above downwards A, B, C, D, and the corresponding prints a, b, c, d. Then, allowing for the perspective inclination of the lines receding at right angles from the plane of the diagram, it will be found that in *form* a is the same as C, b is the same as D, c is the same as A, and d is the same as B, each as each. In other words, in radiography, as in other graphic images, the other side of a picture is not a picture of the other side of the object portrayed ; the other side of a picture is a picture in perspective with a reversed object—an object reversed as in a mirror—and with the same aspect of it presenting to the observer in each case. And the same principles must apply to all parts of the human body, *e.g.* a right hand or a left foot, or to the heart normally situated on the left side of the chest. It is a good mental gymnastic to mentally substitute a right or a left hand with back or palm turned to the radiographic centre for the corresponding cubes of the diagram ; in the case of the normally-situated heart we would only have two cases, as the breastbone or the spine were turned to the photographic plate ; the prints (black on white) would then represent an imaginary normal dextrocardia, pathological dextrocardia being a different thing entirely. As regards *form* therefore, A, B, C, D are direct images in perspective with the corresponding objects, while a, b, c, d are the corresponding reverse images. We now can understand what these reverse images (like X-rays prints viewed from their face, or like the fluorescent screen or X-ray glass plate viewed from the side remote from the focus tube) represent really ; these and the reverse or back of any picture are pictures of a reversed, it may be an unreal, object. What the observer thinks he sees is quite another question.

Such an ideal object as that employed in previous diagrams is of course quite exceptional. As a rule, the object we have to X-ray for diagnosis in practice is superficially visible, with its surface markings and recognisable landmarks, while in the mass opaque to light. Only the side nearer the station point of the eye can be seen therefrom. As regards X-rays, the mass is mainly transparent, so that only parts opaque to X-rays are shown up. The problem is to combine correctly as regards direction the graphic picture of the near side with the radiographic pictures of the interior. We may draw a cubical box with its superficial markings, the box being opaque to light, while it is materially strengthened inside by various bars and rods which are more or less opaque to X-rays, and its contents are supposed to be two air cushions and a bag containing small shot. In diagram VI such an object is shown as taken from one side, and in VII as taken from the other. The combination of the graphic and diagraphic image may be made by ordinary photographic or graphic methods, either on the diagraphic or on the graphic plane as indicated; of course, the latter plan is far and away the most natural and convenient one. I believe myself some form of camera lucida would be of great value in bringing the radiographic image on to the selected surface of delineation, using the necessary precautions to secure direct images only; then at last we would have succeeded in taking sights through the keyhole.

I have ventured in the VIIIth diagram to show experimentally (by copying to scale a frozen section of the human chest at the level of the 7th or 8th dorsal vertebra), among other things the possibility of applying these principles to the human body. The two graphic planes are shown as drawn by hand, they are not really photographic reductions. The bullet shown on the right side of the chest about the tip of a transverse process of a vertebra is also ideal.

The next step must be the clinical one. I do not now expect ever personally to have either time or opportunity to make that farther step; like the sign-post, I can only point the right direction. With a few brilliant exceptions, clinical radiography on wrong lines must be largely futile.

There remains the unfortunate position of the votaries of the fluorescent screen, which is only, after all, a kind of view finder. With the screen the medical radiographer is like the tourist who found himself in the wrong train, while his luggage remained in the right train, or like "Alice through the Looking Glass." Yet he might get back (by some much more simple apparatus than the ortho-diagraph) by the use of appliances everywhere at hand. In fact, for him could be visualised the radiographic centre.[3]

O, then, is the centre of all our diagnostic hopes. How is it to be recorded, and how reproduced ? One simple means is some apparatus that really is a radiographic camera, which I have provisionally called the "camera (perhaps better antecamera) aperta." Details will be found elsewhere. The principle is to keep the radiographic centre always perpendicularly opposite the geometrical centre of the screen or other X-ray picture.

Unfortunately for our profession Röntgen was not like Helmholtz a medical man. The initial error was made by the German physicians and surgeons. It is not difficult to see how it has arisen and even how it has persisted. But once the medical radiographers are convinced, everything will come right, and radiography will take its proper clinical position, and cease to be so much a happy hunting ground for the mechanical engineer.

There are at least three simple ways by which direct X-ray images in perspective with the object can be obtained correct both in form and in matter of tone, namely with dark shadows and white high-lights ; but the adoption of any such plan while the present paradox is still paradox (awaiting promotion to heresy) would only lead to confusion worse confounded. As at present, then, let white on black remain the X-ray negative viewed from the film side, and let black on white remain the X-ray print viewed from its face—until, in short, paradox becomes orthodox. But I would appeal to all editors and printers that they will be kind enough to leave the backs of their X-ray reproductions unprinted on, so that the X-ray print—black on white—can always be held up to the light, and viewed through its back by the increasing number of medical radiographers who will take the same point of view as myself.

One can do very much what one pleases with straight lines upon a large drawing-board, and with great accuracy : and many facilities are afforded to the experimenter in the absence of unsteadiness and flickering, and in the possibility of spacing one's diagraphic plane well away from the object.

I have to thank Professor Barrell, of Bristol University, for

Diagram VI.

Diagram VII.

Diagram VIII.

revising the mathematical part of this paper, and Mr. W. H. Hawker, Bristol, for his photographic reductions of the original diagrams.

BIBLIOGRAPHY.

[1] "X-Ray Photographs as Pictures," *Bristol M.-Chir. J.*, 1901, xix. 131.

[2] "The Essential Ambiguity of X-Ray Representation, and Some Methods of Solution," *J. Röntgen Soc.*, 1910.

[3] "The Fluoroscopic Diagnosis of Direction by a Plane Mirror upon the Screen," *Practitioner*, 1911, lxxxvi. 725.

[4] "Design for an Elementary Radiographic Camera," *Bristol M.-Chir. J.*, 1911, xxix. 118.

---

## Figures and Tables

**Diagram I. f1:**
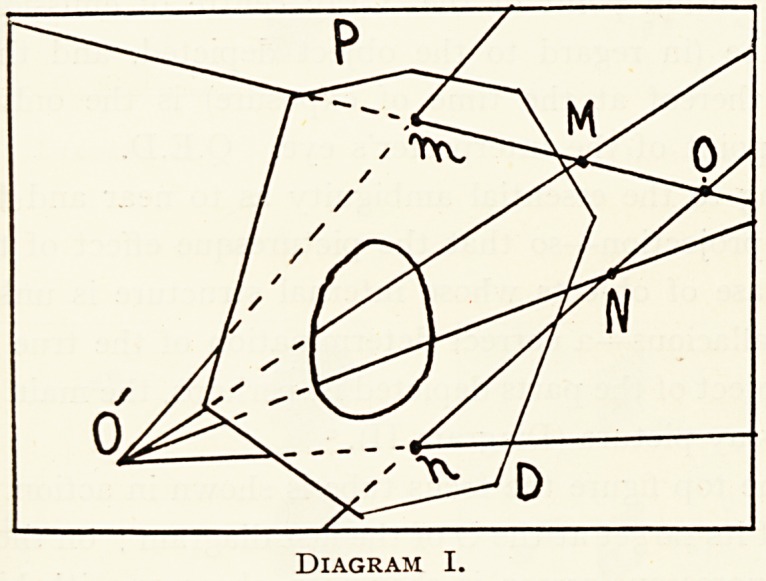


**Diagram II. f2:**
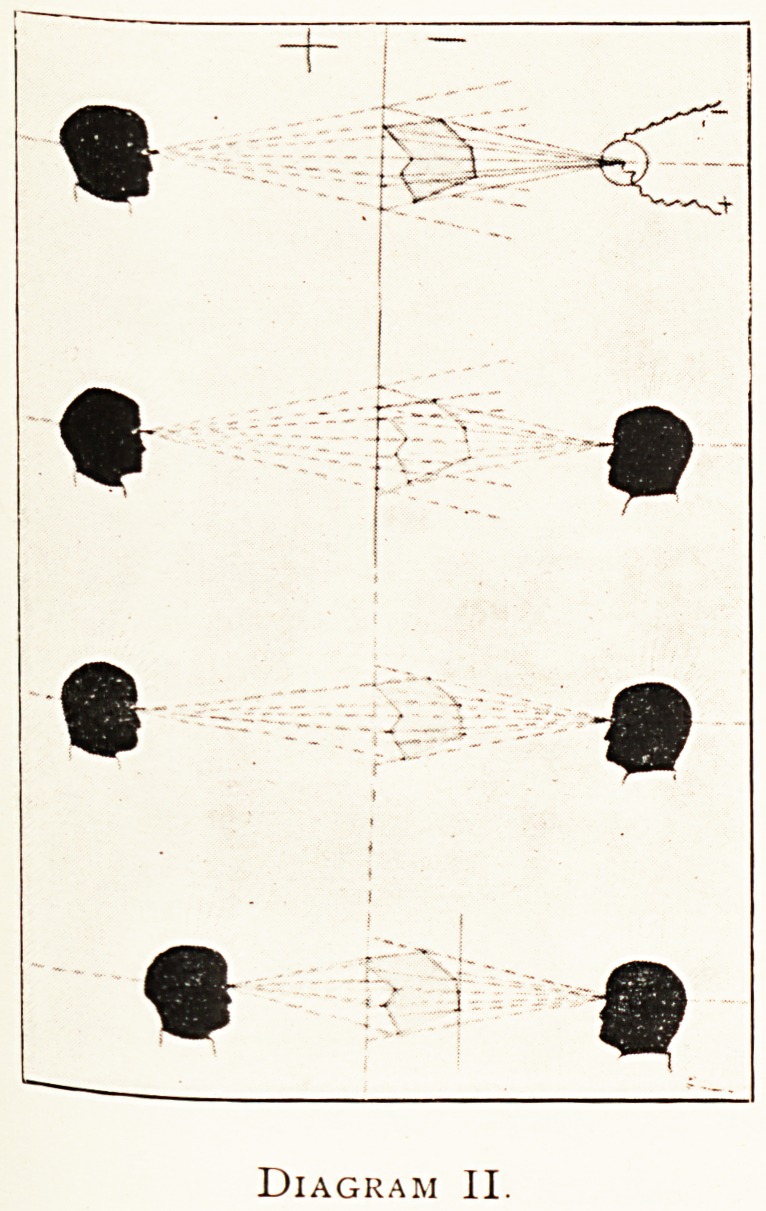


**Diagram III. f3:**
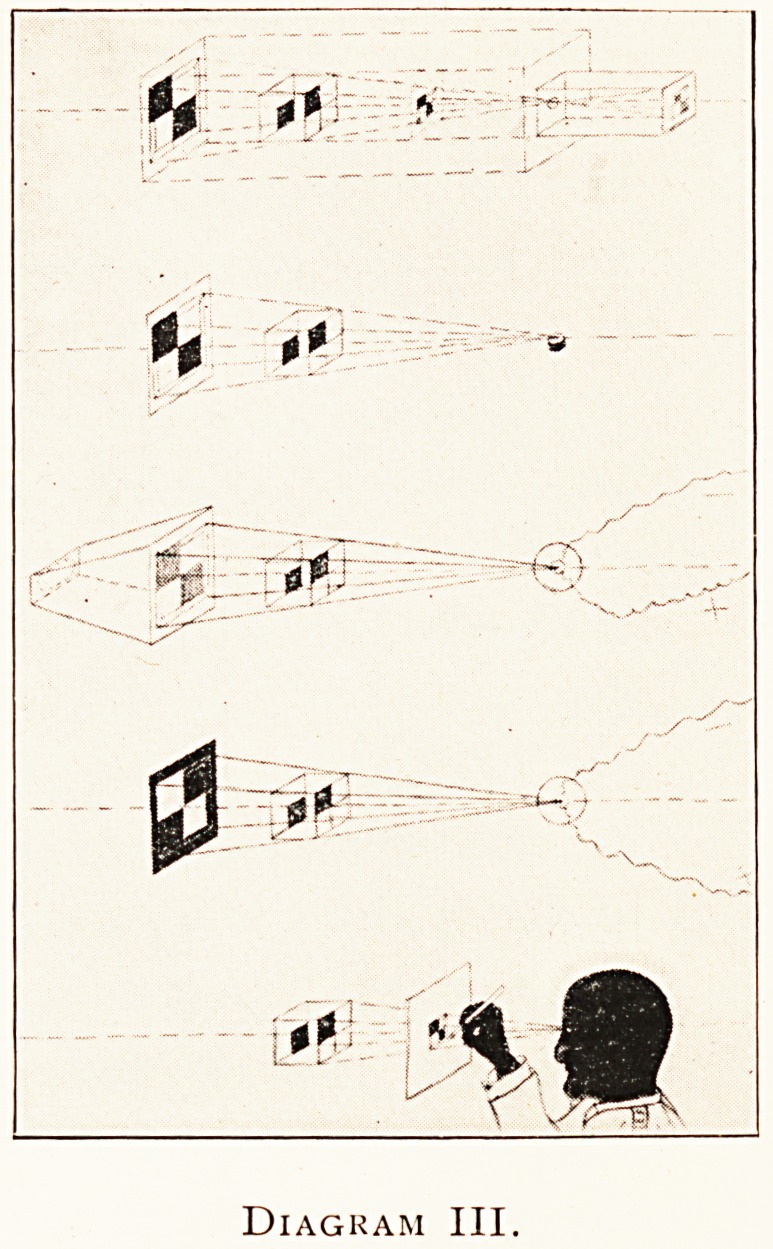


**Diagram IV. f4:**
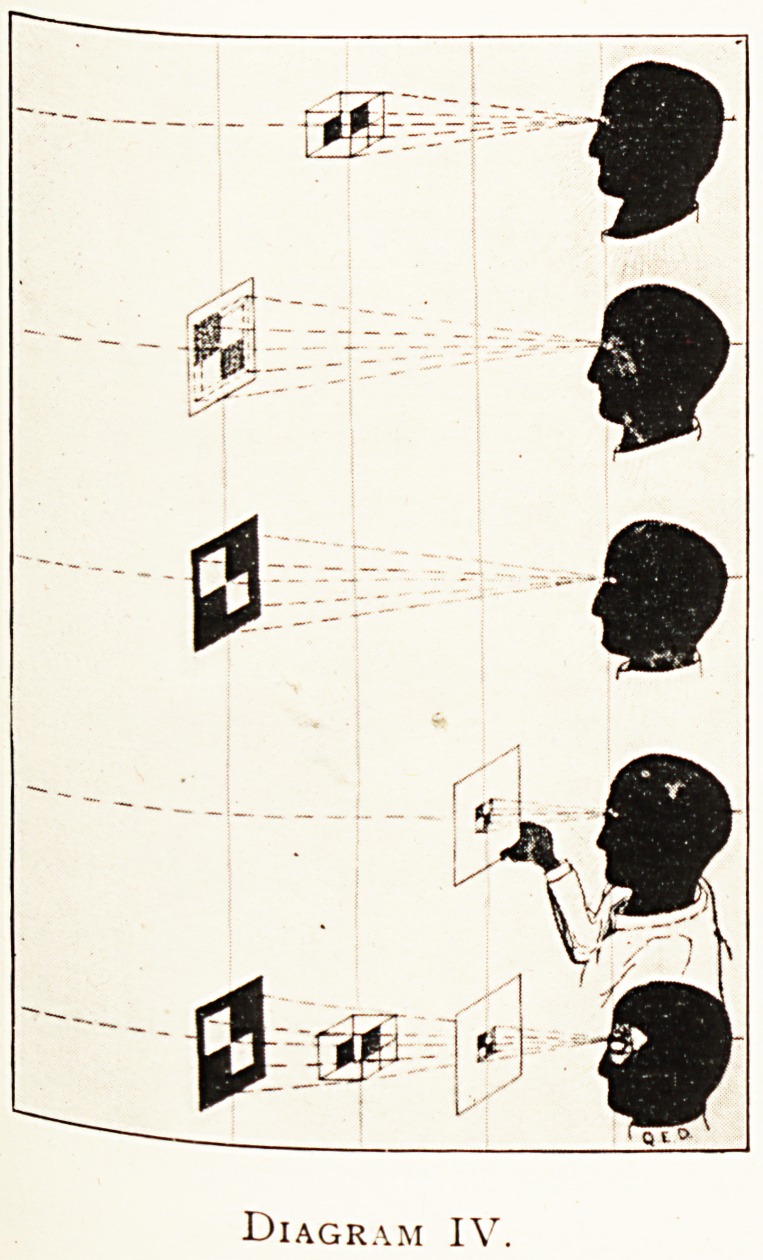


**Diagram V. f5:**
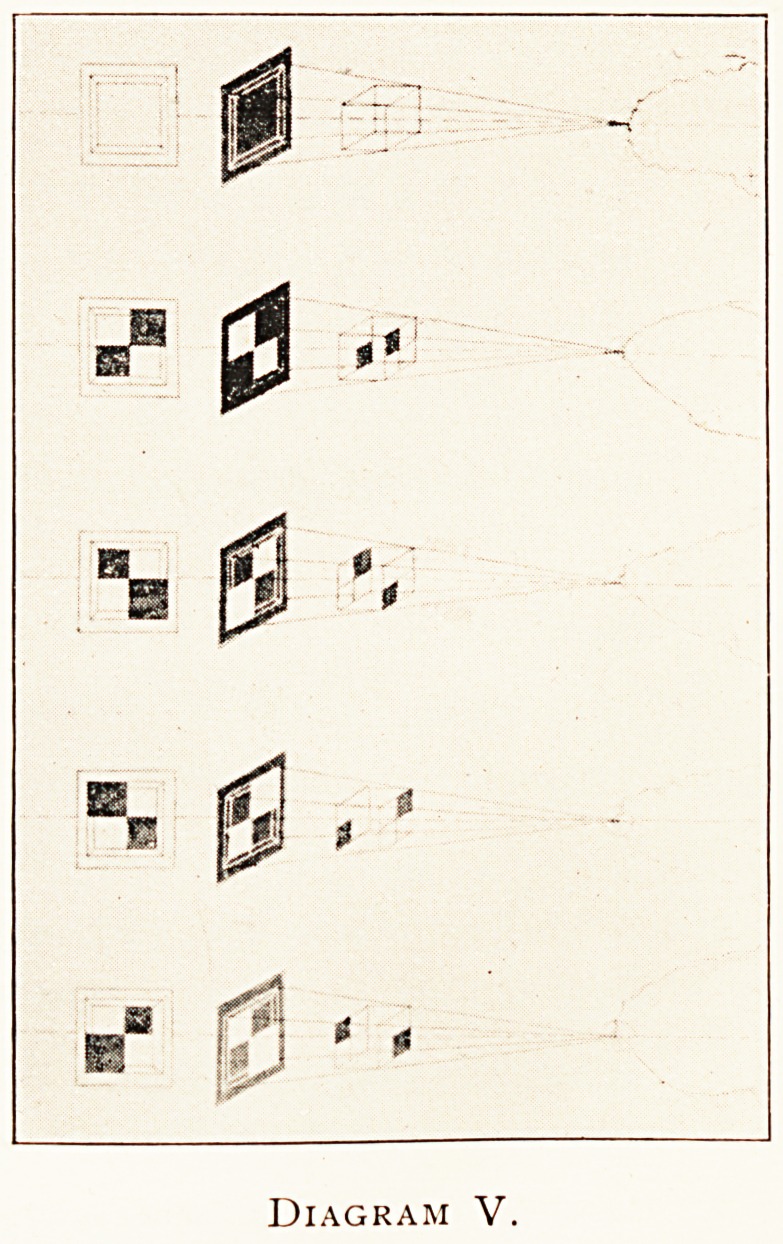


**Diagram VI. f6:**
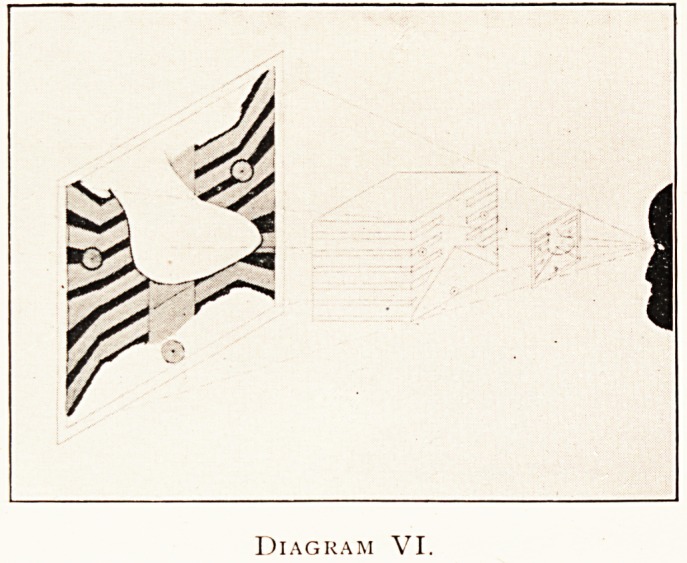


**Diagram VII. f7:**
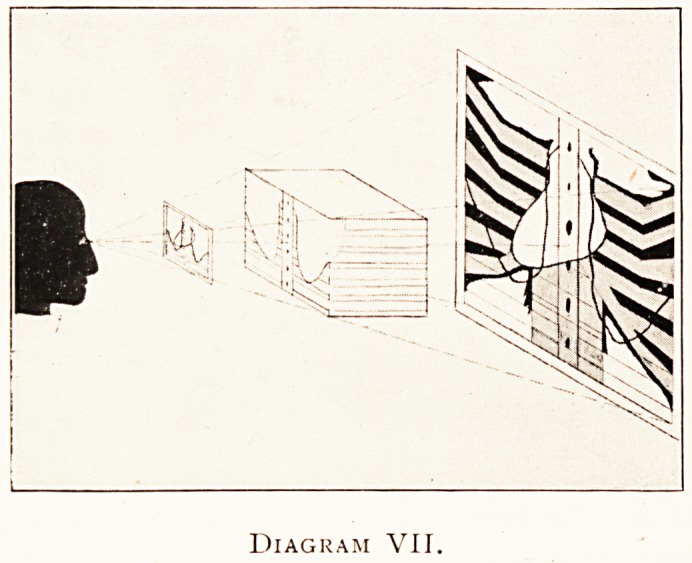


**Diagram VIII. f8:**